# Computer Vision Classification of Barley Flour Based on Spatial Pyramid Partition Ensemble

**DOI:** 10.3390/s19132953

**Published:** 2019-07-04

**Authors:** Jessica Fernandes Lopes, Leniza Ludwig, Douglas Fernandes Barbin, Maria Victória Eiras Grossmann, Sylvio Barbon

**Affiliations:** 1 Department of Computer Science, Londrina State University (UEL), Londrina 86057-970, Brazil; 2 Department of Food Sciences, Londrina State University (UEL), Londrina 86057-970, Brazil; 3 Department of Food Engineering, University of Campinas, Campinas 13083-970, Brazil

**Keywords:** machine learning, image processing, food quality, computer intelligence

## Abstract

Imaging sensors are largely employed in the food processing industry for quality control. Flour from malting barley varieties is a valuable ingredient in the food industry, but its use is restricted due to quality aspects such as color variations and the presence of husk fragments. On the other hand, naked varieties present superior quality with better visual appearance and nutritional composition for human consumption. Computer Vision Systems (CVS) can provide an automatic and precise classification of samples, but identification of grain and flour characteristics require more specialized methods. In this paper, we propose CVS combined with the Spatial Pyramid Partition ensemble (SPPe) technique to distinguish between naked and malting types of twenty-two flour varieties using image features and machine learning. SPPe leverages the analysis of patterns from different spatial regions, providing more reliable classification. Support Vector Machine (SVM), k-Nearest Neighbors (k-NN), J48 decision tree, and Random Forest (RF) were compared for samples’ classification. Machine learning algorithms embedded in the CVS were induced based on 55 image features. The results ranged from 75.00% (k-NN) to 100.00% (J48) accuracy, showing that sample assessment by CVS with SPPe was highly accurate, representing a potential technique for automatic barley flour classification.

## 1. Introduction

Barley is one of the most ancient cereal crops grown by humanity [1]. Over the years, some barley cultivars (e.g. malting or hulled barley) were selected for the malt and brewery industry, while other cultivars were selected to be used as food ingredients. These last cultivars are known as naked, or even hull-less or uncovered barley, generally containing higher amounts of soluble fiber [2,3]. Requirements concerning barley characteristics are quite different for malting and food industries. For brewery, grains with a low β-glucan concentration and barley kernels with a tough inedible outer hull still attached are required. High β-glucan levels interfere negatively in the malting filtration process. Furthermore, the loss of husks during malting processes leads to a reduction in malt quality. Such characteristics are inherent in hulled varieties [4]. On the other hand, barley cultivars with high levels of proteins and β-glucan (a functional ingredient) are preferred in the food industry, and some further specifications may vary depending on the requirements of each product. As an example, flours from naked types are preferably used for infant foods because they generally have fewer husk fragments [5].

Due to vast applicability, barley is one of the four significant grains, being used for various organic food materials [6,7]. Despite the genetic resource of a variety being the significant factor in determining its technological characteristics, it is well established that environmental conditions and interactions between environment and genotype can modify the expression of such characteristics [8]. Consequently, it is difficult to predict the best industrial destination for barley, or other cereal grains, without performing some physical and chemical analysis, which are generally expensive, time-consuming, and/or require specialized analysts and equipment [9]. The agricultural and food industries are often searching for fast and accurate technologies to increase processing performance, improving product quality. Imaging sensors and computer vision systems have been developed for grading product quality, discriminating among varieties, and detecting contaminants or added substances [10,11,12].

Quality evaluation can be performed by a Computer Vision System (CVS) based on an acquisition device (digital camera, inexpensive and broadly available) and prediction models using machine learning algorithms. This type of approach presents several advantages, including rapidity, low cost, and accuracy and can be applied to grains/seeds [13,14], flours [11], or other agricultural by-products. Being non-invasive methods and not employing chemical reagents, they can be considered as eco-friendly technologies. Product inspection is in high demand in the food industry, including quality inspection, process control, classification, and grading. Manual inspection by visual examination demands a long time and is tedious and inefficient. Machine vision is suitable for this task, as computer vision provides an economical and fast alternative for food processing inspection [15].

The visual aspect is one of the most important parameters for the assessment of food quality. The general utilization of processing equipment in the industry has increased the risk of foreign material contamination [16]. Adulteration, contamination, or simply grading of products according to their visual characteristics are a common need in food processing. For instance, due to the resulting potential health threat to consumers, the development of a fast, label-free, and non-invasive technique for the detection of adulteration over a wide range of food products is necessary [17]. Hence, the food industry is interested in optimizing not only the nutritional characteristics of food products, but also their appearance, including color, texture, etc. It is essential to investigate objective methods that can quantify the visual aspects of food products [18].

To meet the demand for high-quality produce, grains are classified according to their characteristics, before being sent for processing. Manual inspection of in-process products is difficult considering the sampling from processing lines [15]. Considering that barley grains are inhomogeneous, imaging techniques will have extensive practical applicability as analytical tools during industrial processing. Regarding all the chemical-free techniques available, there are still some common challenges before transferring recent research achievements obtained from a laboratory scale to industrial applications, such as building innovative data analysis algorithms that can thoroughly filter redundant information; exploiting appropriate statistical techniques for improving the model robustness for real-time operations; and decreasing the cost of the instrument [19].

In digital image analysis, spatial pyramid methods are very popular for preserving the spatial information of local features, focusing on improving the pattern description [20]. Sharma et al. [21] proposed Spatial Pyramid Partition (SPP) and highlighted that in many visual classification tasks, the spatial distribution carries important information for a given visual classification task. However, the proposed SPP method based on bag of features leads to enlarging the feature vector when several image descriptors take place, resulting in a highly dimensional problem and demanding feature extraction or feature selection methods.

**Figure 1:**
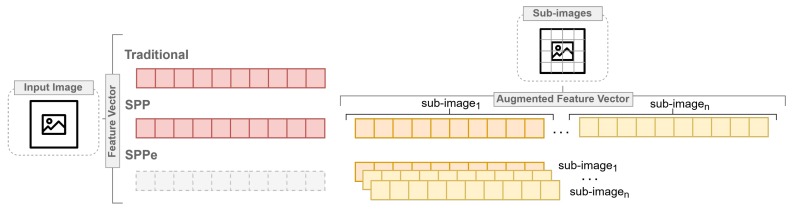
General overview highlighting the differences among traditional, SPP and Spatial Pyramid Partition ensemble (SPPe) approaches of feature vector composition.

Szczypinski et al. [9] classified barley grain varieties based on image-derived shape, color, and texture attributes of individual kernels. Considering barley flour classification, spatial information is required, but the original SPP is not feasible in our problem due to the characteristics of bag of features. In other words, barley grain and flour image-based classification demand several features, while flour image analysis requires more robust pattern recognition approaches than SPP can provide. However, the increase of dimensionality arising from the application of SPP is a challenge in the machine learning scenario, and consequently for CVS applications. Therefore, we proposed the Spatial Pyramid Partition ensemble (SPPe), an ensemble technique fashioned on SPP towards supporting a suitable image pattern description in scenarios with a considerable number of features, as exposed in Figure 1.

A vast number of characteristics might rely on performance improvement of prediction tasks. The traditional feature extraction method considers the whole image at once for extracting its features, which possibly decreases important spatial information of some image descriptors from samples. As previously mentioned, the SPP was proposed to improve the problem task that requires some localized descriptors. However, it is based on a bag of features grounded on splitting images into sub-regions for supporting additional spatial information. Thus, SPP appraises a visual descriptor vector composed by the original image and its sub-regions from each sample.

The proposed SPPe was evaluated in a CVS with a set of image features based on color, intensity, and texture, in comparison to SPP [21], directly using the features extracted from the Region Of Interest (ROI), as traditional CVS [13,22,23,24]. We compared the performance of four different machine learning algorithms: Random Forest (RF), Support Vector Machine (SVM), k Nearest Neighbor (k-NN), and J48 decision tree for modeling the classifier. These algorithms were employed to distinguish between naked and malting barley flour with image features extracted from 22 varieties acquired from five samples of each variety.

## 2. Related Work

Several studies presented CVS with machine learning methods applied to improve the prediction of a given parameter. Some CVS require sophisticated modeling to cope with non-linearities and noisy and imbalanced datasets. The application of Machine Learning (ML) techniques for food attributes’ prediction and quality evaluation has been widely investigated [10,22,25,26,27,28,29,30]. ML can be applied to extract non-trivial relationships automatically from a training dataset, producing a generalization of knowledge for further predictions [31]. Hence, machine learning promotes high performance as an alternative for an intensive agricultural operational process of the agri-technologies domain [32].

Random Forest (RF) [33], Support Vector Machine (SVM) [34], k-Nearest Neighbors (k-NN) [35], and the J48 decision tree algorithm [36] are well-established machine learning algorithms applied in many studies related to food quality analyses. RF was compared to SVM for an automated marbling grading system of dry-cured ham [37]. The SVM algorithm showed better performance with 89% of the samples correctly classified. Another application of SVM was described in Papadopulou et al. [27], achieving over 89% of accuracy for classification of beef fillets according to quality grades. For analyzing image features to evaluate the impact of diets on live fish skin, Saberioon et al. [38] applied four different classification methods, and SVM provided the best classifier with 82% of accuracy. Barbon et al. [23] proposed a CVS for meat classification based on image features, managed by an instance-based system using k-NN to classify meat according to marbling scores from image features. The authors presented an accuracy of 81.59% for bovine and 76.14% for swine samples, using only three samples for each marbling score by the k-NN prediction models. Granitto et al. [30] applied RF for the discrimination of six different Italian cheeses. In addition to reasonable accuracy, the RF model provided an estimation of the relative importance of each sensory attribute involved. The effectiveness of RF was also highlighted in a CVS used for predicting the ripening of papaya from digital imaging [22].

Considering barley applications, Nowakowski et al. [39] evaluated malt barley seeds using four barley varieties. The feasibility of image analysis was applied with machine learning and morphology and color features, achieving 99% accuracy. Kociolek et al. [40] classified barley grain defects using preprocessed kernel image pairs for feature extraction based on morphological operations. Pazoki et al. [41] identified cultivars using rain-fed barley seeds. The proposed method was applied with 22 features extracted from three varieties of samples, which fed a Multilayer Perceptron (MLP). The features of color, morphology, and shape were used for individual rain-fed barley seeds. Different network architectures were explored, including feature selection, resulting in 82.22% accuracy. Ciesielski and Nguyen [42] proposed to distinguish three different classes of bulk malt (made by barley grains). Image texture features were extracted and classification was performed with k-nearest-neighbor (k-NN), achieving an accuracy of 77.00%. According to the authors, the classification through the evaluation of individual kernels is time-consuming, and many kernels are required to obtain a significant estimation of the modification index from a whole batch. Nevertheless, separating the samples in minimal milling portions is a booster alternative, aiding the evaluation of the difference between barley types. Lim et al. [7] explored Near Infrared Spectroscopy (NIRS) and a PLS-DA discrimination model to predict hulled barley, naked barley, and wheat contaminated with *Fusarium*. The authors achieved high accuracy at the cost of the complexity of NIRS equipment and signal processing.

Accordingly, the above studies have performed image analysis at different stages for varieties’ identification for industrialization and improvement purposes. Integrating the industrial environment promotes a major role for developing an automated system for distinguishing agricultural raw-material products. The approach introduced in this paper is a CVS with an adaptation of the original SPP, modifying the overview perspective of sub-images that compose an original sample. The proposed approach is based on splitting each image into several sub-regions to predict a respective sample. We propose a method to improve prediction performance using CVS with machine learning, by applying the SPPe technique.

## 3. Materials and Methods

Twenty-two different barley varieties (cultivars) were provided by EMBRAPA Trigo (Brazilian Agricultural Research Corporation) in the city of Passo Fundo (Brazil). Barley samples were dehulled (Codema Inc. equipment, Maple Grove, MN, USA) during 75 s and milled (IKA A11 Basic Micro Miller, Osaka, Japan) for 75 s. Five different color images of flour were acquired from each of the 22 cultivars, in a total of 110 samples. Samples were collected and labeled by a specialist according to the source types of barley. After, all samples were classified either as malting Barley (B) or Naked barley (N). Fourteen of the cultivars were identified as malting barley, and eight were naked barley. Letters are followed by numbers in order to indicate differences from each specific barley variety (Table 1).

### 3.1. Computer Vision System

**Figure 2:**
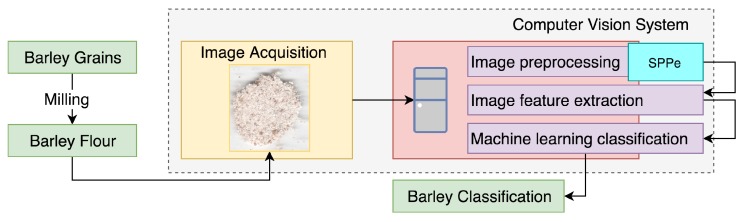
General overview of the proposed approach.

The CVS was constructed to classify samples as malting or naked barley, through the analysis of barley flour images. The employed CVS can be detailed as four main steps: acquisition, preprocessing, feature extraction, and classification (Figure 2).

It is important to highlight that the proposed SPPe is a technique to improve the classification performance grounded on a more informative strategy from the image sample before image feature extraction. SPPe requires interactive production of sub-images from an original sample image. These new sub-images had features extracted for enriching the dataset with complementary sources of information. Prediction of the original sample was based on a voting process for the sub-image samples’ classification, as detailed in Section 3.2.

#### Image Acquisition and Preprocessing

**Figure 3:**
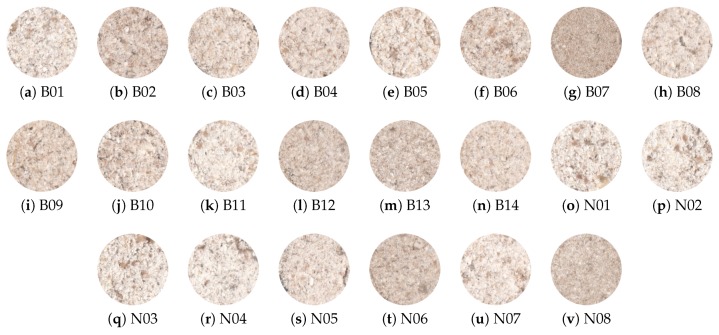
Samples of barley flour from malting (**a**–**n**) and naked (**o**–**v**) types.

The samples were collected from two different types for creating the image dataset: malting barley B and naked barley N (Figure 3).

Images (1200 dpi) were acquired by a computer vision system where each individual barley flour portion was scanned (HP Laser Jet M1120 MFP, Hewlett-Packard, Louveira, Brazil), using image acquisition software (HP Precision Scan Pro, Version 6.1, 2009). The images were acquired (14,028 × 10,208 pixels) and stored as a .jpg file for further processing, as described in Section 3.2.1. A total of 110 barley sample images were collected, five from each cultivar, 40 from naked barley and 70 from malting barley. The ROI was cropped from the original image considering the largest square in individual portion of barley flour, removing the background and contour of each sample.

The main goal of preprocessing was background removal, keeping the ROI. To achieve this, the image was converted to the monochromatic space channel, and the background was removed using image thresholding. This threshold value was selected using Otsu’s thresholding since it is one of the most widely-used methods for image segmentation. Since this image thresholding may lead to the removal of some pixels of the ROI, all the holes in the barley flour area were filled using a connectivity approach. At this point, the obtained image mask (representing the foreground) was used to find the center of mass of the object (barley flour samples). As the final step, the center of the mask found was used to grow a predefined square until reaching the object edge. The square mask was applied on to the original image, cropping the ROI.

### 3.2. Spatial Pyramid Partition Ensemble

In the current work, we propose SPPe as part of the preprocessing step (Figure 2), to obtain a complete pattern comprehension of each sample. Our technique is a modification of the traditional SPP proposed in Sharma et al. [21]. Spatial Pyramid Partition (SPP) is based on splitting each image into a sequence of smaller sub-regions, extracting local image features from each image, and encoding their features into a vector [43,44]. In this sense, a given image is viewed as its low-level visual features extracted from all sub-regions. Each image is split into three levels, Level 0 being the image of the ROI by removing edges; Level 1 subdivides the ROI into four distinct parts, extracting its features; Level 2 subdivides each of the previous partitions into four other partitions, totaling 21 images from each ROI for extracting features to fine-tune the dataset. As a result, high-level and low-level features are extracted from the SPP image sequence to compose the image feature vector [21].

**Figure 4:**
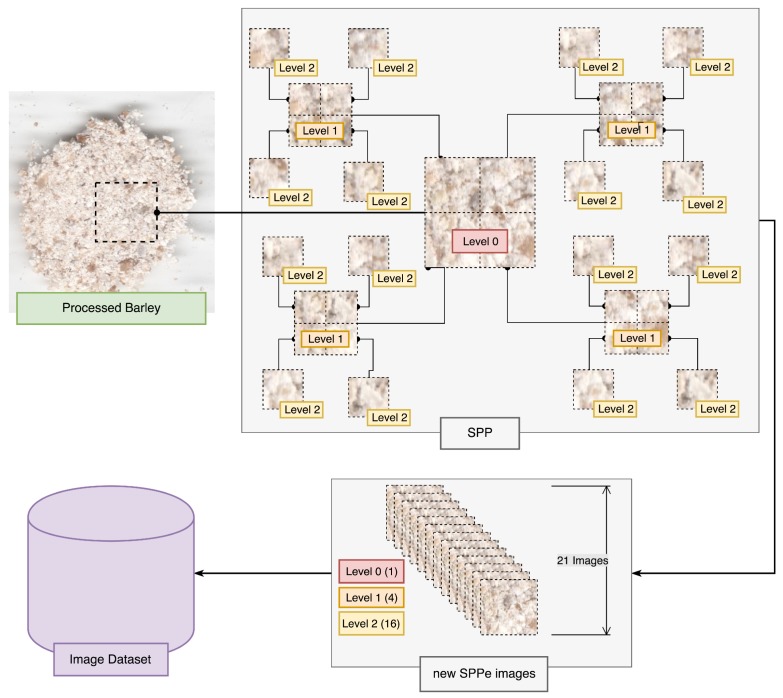
Spatial Pyramidal Partition ensemble (SPPe) for obtaining image samples.

The proposed SPPe adapted the original SPP using an ensemble strategy to obtain the image classification. As opposed to traditional SPP, the aggregated image feature vector was not comprised of all sub-region, as a bag of features. Figure 4 presents an overview of the SPPe approach of creating sub-regions. Considering the description of image splitting, a new dataset was formed, which was composed of the sub-regions designated as Level 1 and 2. Thus, a feature vector was built from each sub-region without concatenating all regions. The ensemble strategy was applied to modify the dataset samples made up of smaller regions (Figure 4). Therefore, the sub-regions were used for problem modeling. After the prediction of a given sample from each sub-region, the scheme applied a weighing vote. In other words, we employed SPP with a subdivision strategy, to classify the Level 0 samples, and we considered each image separately for classification. Following the sub-regions’ prediction, we aggregated them with the respective sample to analyze as Level 0. In this way, from a new sample image, each sub-region obtained from SPPe was classified, and the final decision was achieved by a voting step.

A single model was induced for predicting all sub-regions from different levels. The induction of the classification model was carried out in the Leave-One-Subject-Out (LOSO) scheme to avoid bias [45]. The method employed the LOSO procedure to bind the sub-regions and their image Level 0, keeping all of them together in the training or test phase. In other words, each sub-image was bound to the respective sample (Level 0) and received its label. Hence, the sub-regions were considered non-independent regions as part of the same sample. This methodology guarantees the model learns nothing about the subject to be predicted. Thereby, the technique to be applied decreases the learning bias, achieving accurate results.

The SPPe output is based on the relation between the number of correct and incorrect sub-regions classified toward a majority decision as an ensemble prediction. Each level of partition by the SPPe method was assigned a voting weight. In the proposed experiment, for Level 1, it was assigned a weight of 1/3 and for Level 2, 1/12 for each ensemble member (image prediction). At the end of the iterations, the final result was computed considering each vote multiplied by the assigned weight. The final classification was obtained as the majority weighted vote from 20 sub-regions (4 from Level 1 and 16 from Level 2). This procedure creates a more reliable source of image classification by reducing overfitting, providing a robust description of barley based on several regions and dimensions.

We performed the original SPP proposed by Sharma et al. [21] in order to compare the SPPe performance improvements. SPPe avoids the high dimensional drawback, as in our scenario, SPP demands a total of 1155 image features per sample, while SPPe maintains only 55, both using only one classification model. Another important factor is related to the presence of visual components (e.g., husks) that could lead to noisy or biased features in the image description vector. Using an ensemble technique such as SPPe, we could reduce the overfitting of the final model [33], since the visually undesired components are lost in the final decision by a minority vote.

Each image obtained from the SPPe method had its features extracted independently of the level by the same descriptors for further analysis (as described in Section 3.2.1).

#### 3.2.1. Image Analysis and Feature Extraction

Step 2 is related to the image feature extraction in a sequence of previous procedures (Figure 2). The extracted features are groups of discriminatory properties suitable to distinguish the classes between naked and malting samples. We extracted a set of 55 image features based on color, intensity, and texture. The list including all image features used in our solution is presented in Table 2.

Concerning color descriptors, statistical moments from the CIE L*a*b* and HSV color spaces were used, similarly to Li et al. [43] and Campos et al. [46]. The image acquired was stored in the RGB format, where each pixel is based on three color space: R (red), G (green), and B (blue). Due to the brightness information presented in the whole color channel from RGB, a good practice is related to selecting a different color space able to isolate brightness. For this reason, the transformation of input images from RGB to CIE L*a*b and HSV was considered toward extracting color features. The CIE L*a*b* and HSV color spaces were explored in this study: L* (Lightness), a* (red-green), b* (yellow-blue), Hue (H), Saturation (S), and Value (V) color channels, respectively. The mean and standard deviation were calculated for each color channel. Moreover, we computed the standard deviation, kurtosis, and skewness from the histogram of each channel, comprising a total of 30 color features.

Likewise, the same five statistical moments were used to describe the intensity information of each image. The pixel intensity was calculated from the average of RGB values. Image entropy, which can be characterized as a statistical measure of the randomness, texture, and contrast of grey scale images, was calculated for the intensity channel [47].

Both color and intensity variations between samples can be observed in Figure 3. Therefore, those features were used to properly describe the samples, allowing the machine learning algorithms to find the correct relations between features and barley types.

The texture is an important feature to identify objects or the presence of patterns in an image [48]. In this case, texture features were used to distinguish between different types of barley. For example, the presence of husk fragments in milled barley affects some features and could characterize a specific type of barley flour. Thus, having general applicability, three texture descriptors were used: Local binary patterns [49], Grey Level Co-occurrence Matrix (GLCM) [48], for which distance d=1 and angle $0^{\circ }$ considering 256 grey levels, and Fast Fourier Transform (FFT), this last to uncover frequency domain characteristics [50,51].

It is important to mention that we selected some traditional image descriptors to compose our feature vector, leveraging the comparison among the approaches for barley flour classification. Nevertheless, different image classification tasks can take more advantage of SPPe by employing alternative image features, e.g., features grounded on discrete wavelet transform [52] or fractal dimension [53].

#### 3.2.2. Machine Learning

Features extracted from images are often used for classification and regression models, in order to identify samples from different classes or to predict quality parameters. In this way, machine learning algorithms can induce models from image features for automatic classification of barley flour. The modeling complexity of a machine learning system can vary greatly, allowing a high degree of customized freedom with appropriate trade-offs inherent in each specific scenario [54]. Some of the approaches include linear methods and non-linear machine learning algorithms, such as k-nearest neighbor, support vector machine, J48 decision tree, and random forest [46].

A brief description of the algorithms and the corresponding packages used to implement each ML algorithm are described in Table 3. In our experiments, the hyperparameters used were the default values of Rpackages in order to support a fair comparison among the algorithms.

In our experiment, the algorithms were applied in the R environment to induce models for barley flour classification. In order to achieve a reliable evaluation, two datasets were created: cross-validation and prediction test set. The cross-validation set was used to induce the models, adjusting the hyperparameters 10-fold considering 1800 images (Levels 1 and 2), while the prediction set was employed to test the classification performance using 400 images (Levels 1 and 2). Separation of samples into training and test sets was made in order to minimize the risks of overfitting, using the Kennard–Stone algorithm [60]. It is important to mention that the samples were split into the training and testing set considering Level 0 (a group of sub-regions), 90 samples (81.8%) for training and 20 samples (18.2%) for testing.

### 3.3. Evaluation Metrics

Performance evaluation of the models from machine learning was done using the total accuracy method (accuracy matrix) [61]. It is computed through the confusion matrix, which is defined by Equation (1).

(1) $$Total\;Accuracy=\frac{TP+TN}{n}$$

The total accuracy is calculated by the sum of the main diagonal values from the confusion matrix. These values are the True Positive (TP) and True Negative (TN), which are divided by the sum of the values from the whole matrix (*n*). Thus, it is possible to compute the performance of the image features and machine learning algorithms through the relation of the correctly-classified samples of barley flour. Recall (Equation (2)) and precision (Equation (3)) are often used to evaluate the effectiveness of classification methods based on False Negatives (FN) and False Positives (FP). In our work, we employed these metrics in order to support a fair comparison of the methods’ quality.

(2) $$Recall=\frac{TP}{TP+FN}$$

(3) $$Precision=\frac{TP}{TP+FP}$$

Additionally, processing time from feature extraction to prediction was compared. Thus, it is possible to estimate overall job execution with an additional perspective of performance analysis. In the experiments, the time cost was calculated as the average of 30 runs. Dealing with descriptors, random forest importance was applied to this approach. The RF algorithm estimates the importance of a variable being observed when the prediction error increases if data for that variable are permuted, while all others are left unchanged. Based on the trees, as the random forest is constructed, RF’s importance investigates each extracted image feature, measuring the impact of characteristic samples in order to predict them [33].

In order to evaluate features extracted from barley flour samples, the exposed metric of variable importance demonstrates the advantage of random forest permutation because it embraces the impact of each predictor variable individually, as well as in multivariate interactions with other predictor variables.

## 4. Results and Discussion

### 4.1. Algorithms and Image Processing Methods

The results of algorithm performance for the classification of naked and malting barley flour revealed the advantages of the proposed SPPe method, in comparison to SPP and traditional approaches. The experiments showed distinct performance values achieved with the techniques applied to this approach using machine learning algorithms. In order to establish a practical performance testing environment, the experiments were executed with Intel^®^Core i7-6700 CPU 3.40 GHz 16 GB memory. Table 4 summarizes the results obtained for prediction algorithms over the datasets considering performance measures such as: accuracy, precision, recall, and average processing time.

Comparing the machine learning algorithms, k-NN provided the worst performance, with accuracy values equal to or below 80.00% for prediction using all methods investigated. Concerning only the results of the traditional CVS approach, (without SPP or SPPe) for the prediction set, RF obtained superior performance, with 90.00% of accuracy and precision/recall values of 86.67%, while SVM and k-NN presented similar accuracy (80.00%).

The original SPP presented superior results compared to the traditional method. SVM (92.00%) and RF (91.00%) reached superior results compared to J48 (88.00%) and k-NN (70.56%) for accuracy considering the cross-validation set. For the prediction set, RF obtained superior results, similar to SVM (95.00%). The worst metrics evaluated for the prediction set using the SPP technique was k-NN (60.00%), followed by J48 (85.00%).

An improvement of classification accuracy was obtained by SPPe technique with ML algorithms (Table 4). The average performance of classification considering all machine learning algorithms was improved from 83.75% in the traditional method and original SPP to 91.25% in accuracy for prediction sets. It is important to highlight that J48 stood out with 100% accuracy, and k-NN maintained the lowest performance with 95.56% (cross-validation set) and 75.00% (prediction set). Likewise, the SPPe solution had the lowest processing time cost in comparison to SPP. Furthermore, traditional CVS provided better results than SPPe using k-NN.

Considering the processing time of the applied methods, CVS spent less time, being faster than SPP and SPPe, as expected. When comparing SPP and SPPe, our proposal was faster than SPP in all experiments. It is clear that the time cost tends to enlarge when the feature vector expands, as proposed by SPP and SPPe; however, the trade-off between predictive performance and processing time suggests the SPPe as a suitable solution when the main goal is the classification performance.

### 4.2. Evaluation of Image Features

**Figure 5:**
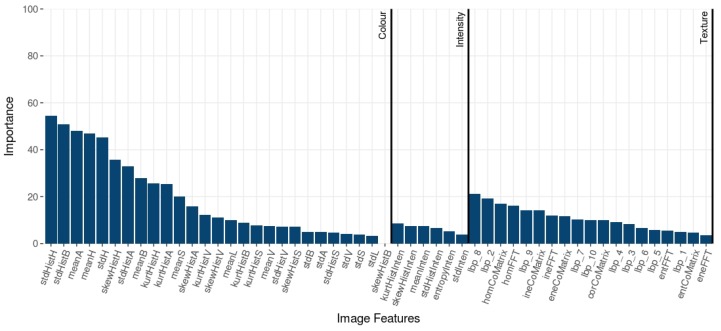
RF importance of image features.

The RF importance exposes the most relevant features in prediction tasks. The importance values are summarized in Figure 5. The most important features were from color: standard deviation values of the H and b* channels histogram (hue from HSV and yellow-blue CIE L*a*b* color spaces) were the most relevant explaining features with more scores higher than 50. Several statistic values from H, B*, and a* overcame texture and intensity features, although all features presented an impact for the classification procedure.

In order to characterize the types of barley flour, the mean and standard deviation of the grey-scale image, and hue HSV channel were the most discriminative image features. Moreover, the values of a* and b* channels gained higher importance, as well as the saturation. Texture features were significant for predicting the samples. Indeed, some texture features of the grey level co-occurrence matrix, and some LBP metrics were efficient at predicting variations of samples and also could be related to the granularity present in the barley flour.

**Figure 6:**
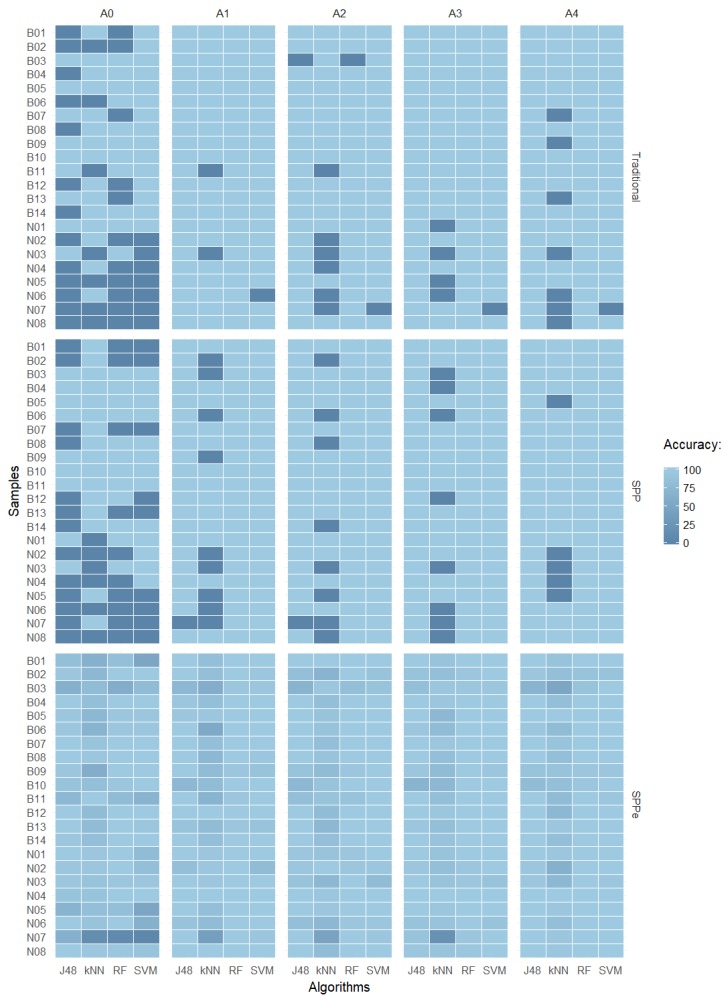
Accuracy heat map of J48, k-NN, RF, and SVM over the prediction dataset comparing traditional CVS, SPP, and SPPe techniques with repetitions A0, A1, A2, A3, and A4.

Figure 6 summarizes the results in which it is possible to observe some misclassified samples by comparing all performed techniques with five different repetitions of acquisition (A0, A1, A2, A3, and A4). Correctly-classified samples are presented in light blue, while dark blue shows the misclassified samples for each method. It is possible to observe that the naked class presented more misclassified samples, meaning it is more complex to predict. For some classification algorithms, it is possible to observe similar behaviors among the samples, with k-NN as the worst performance.

**Figure 7:**
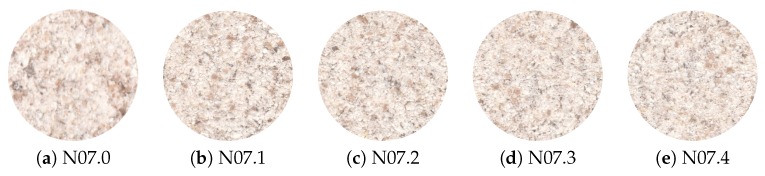
Samples of cultivar N07, the lowest accuracy of barley flour classification.

Analyzing the misclassified samples, it was possible to identify similar patterns in both naked and malting types. Observing the accuracy error, it is possible to conclude that naked sample N07 (37% error) presented similar characteristics to malting samples. Figure 7 presents an overview of the N07 cultivar, where it is possible to observe details by comparing the five samples as previously mentioned, and highlighted in the heat map shown in Figure 6. One possible explanation for the high error rate observed is the fact that Brazilian naked varieties were developed by using malting barley genes, so the studied features can be similar to those of malting barley varieties due to genetic origins [62].

Overall, SPPe demonstrated superior prediction ability compared to other methods, in addition to reduced overfitting and decreasing the high dimensionality present on the original SPP. Differences in composition/physical characteristics between the two barley groups (from naked and malting barley) were detected by the computer vision system, and classification accuracy was improved using SPPe.

### 4.3. SPPe in the Industry

There is an expressive advance when using the SPPe technique in comparison to the traditional CVS and SPP. The best result in the prediction set referred to J48 predictive performance and with low processing time in comparison to SPP. The proposed vision system was designed for an embedded process to provide high-level information for the barley flour industry environment. The system can be implemented by three sub-division steps:

- The input image (acquisition) being extracted from the camera. Images are acquired by a camera placed at the scene under inspection.
- The scene has to be appropriately illuminated and arranged, which promotes suitable reception of the image properties that are necessary for image processing (feature extraction and classification).
- The processing system stage consists of a computer employed for processing the acquired images, resulting in classifying as naked or malting barley flour.

Combining the embedded technology with image processing, a future application in barley flour recognition types for quality control industry is possible.

Our proposal is a viable solution for barley flour industrial processing, as well as similar flour food products. More specifically, our proposal contributes to the industry in different stages of production. The CVS can be used as a quality control, observing specific supplier and providing financial advantages for high-quality flour. The proposed solution can be integrated into processing lines to identify barley according to the application, i.e., whether it is destined for infant formula, health food, and the malting industry, among other industrial production. It is important to highlight that the SPPe was fashioned with a minor feature vector in comparison with the SPP technique, spending less time to process, being faster and promoting its implementation in the production line.

## 5. Conclusions

This work proposed a system based on ML algorithms and computer vision developed to solve the automatic data analysis. A new proposed approach of Spatial Pyramid Partition ensemble (SPPe) provided better results for classification of barley flour into two different classes when compared to Spatial Pyramid Partition (SPP) and traditional CVS. Differences in barley composition cause variation of the flour’s physical characteristics, which were detected by image analysis. The proposed method showed a significant improvement, by reducing overfitting, avoiding dimensional growth, and improving classification accuracy for several machine learning algorithms. The importance of all image descriptors (color, intensity, and texture) for providing helpful information to distinguish between malting and naked barley flour samples was identified. The best model was built using the SPPe with J48 decision tree, allowing the classification of 100% of samples. The results of this study are promising, and they could allow the development of an effective model in order to expand its use in the food industry, reducing costs and improving the effectiveness of automatic quality inspection. 

## Figures and Tables

**Table 1:** Barley cultivars employed in the experimentation.B, malting Barley; N, Naked barley.

Sample ID	Cultivar	Type
B01	BRS Aliensa	Malting
B02	BRS Itanema	Malting
B03	BRS Brau	Malting
B04	MN 6021	Malting
B05	BRS Sampa	Malting
B06	BRS Korbel	Malting
B07	MN 6021	Malting
B08	BRS Elis	Malting
B09	BRS Korbel	Malting
B10	BRS Elis	Malting
B11	BRS Mandurí	Malting
B12	BRS Brau	Malting
B13	BRS Cauê	Malting
B14	BRS Cauê	Malting
N01	149852	Naked
N02	149853	Naked
N03	149857	Naked
N04	149846	Naked
N05	149858	Naked
N06	149841	Naked
N07	149855	Naked
N08	149859	Naked

**Table 2:** List of all image features used in the proposed SPPe approach for barley flour classification.

No.	Type	Name	Description
1	Color	meanH	Mean value of the H channel
2	Color	StdH	Standard deviation of the H channel
3	Color	meanS	Mean value of the S channel
4	Color	stdS	Standard deviation of the S channel
5	Color	MeanV	Mean value of the V channel
6	Color	stdV	Standard deviation of the V channel
7	Color	stdHistH	Standard deviation of H channel histogram
8	Color	kurtHistH	Kurtosis of H channel histogram
9	Color	skewHistH	Skewness of H channel histogram
10	Color	stdHistS	Standard deviation of S channel histogram
11	Color	kurtHistS	Kurtosis of S channel histogram
12	Color	skewHistS	Skewness of S channel histogram
13	Color	stdHistV	Standard deviation of V channel histogram
14	Color	kurtHistV	Kurtosis of V channel histogram
15	Color	skewHistV	Skewness of V channel histogram
16	Color	meanL	Mean value of the L channel
17	Color	stdL	Standard deviation of the L channel
18	Color	meanA	Mean value of the A channel
19	Color	stdA	Standard deviation of the A channel
20	Color	meanB	Mean value of the B channel
21	Color	stdB	Standard deviation of the B channel
22	Color	stdHistL	Standard deviation of L channel histogram
23	Color	kurtHistL	Kurtosis of L channel histogram
24	Color	skewHistL	Skewness of L channel histogram
25	Color	stdHistA	Standard deviation of A channel histogram
26	Color	kurtHistA	Kurtosis of A channel histogram
27	Color	skewHistA	Skewness of A channel histogram
28	Color	stdHistB	Standard deviation of B channel histogram
29	Color	kurtHistB	Kurtosis of B channel histogram
30	Color	skewHistB	Skewness of B channel histogram
31	Intensity	meanInten	Mean value of intensity image
32	Intensity	StdInten	Standard deviation of Intensity image
33	Intensity	entropyInten	Entropy of intensity image
34	Intensity	stdHistInten	Standard deviation of Intensity image histogram
35	Intensity	kurtHistInten	Kurtosis of intensity image histogram
36	Intensity	skewHistInten	Skewness of intensity image histogram
37–46	Texture	lbp_0 - lbp_9	Vector of Local Binary Patterns (LBP) rotationally invariant features
47	Texture	entCoMatrix	Entropy of grey-level co-occurrence matrix
48	Texture	ineCoMatrix	Inertia of grey-level co-occurrence matrix
49	Texture	eneCoMatrix	Energy of grey-level co-occurrence matrix
50	Texture	corCoMatrix	Correlation of grey-level co-occurrence matrix
51	Texture	homCoMatrix	Homogeneity of grey-level co-occurrence matrix
52	Texture	eneFFT	FFT Energy
53	Texture	entFFT	FFT Entropy
54	Texture	ineFFT	FFT Inertia
55	Texture	homFFT	FFT Homogeneity

**Table 3:** Machine learning algorithms used in the experiments and corresponding R packages.

**Algorithm**	**Description**	**R Package**	**Hyperparameters**
K-Nearest Neighbor (k-NN)	A non-parametric lazy learning algorithm; the training data are not used for any generalization [55].	RWeka	Euclidean distance; k= 5
Decision Tree (J48)	A decision tree widely applied to represent series of rules that lead to a class or value [56,57].	RWeka	C = 0.25; threshold = 0.25; with pruning
Random Forest (RF)	A combination of decision tree models that provides more accurate prediction [33,58].	RandomForest	ntree = 100; mtry = 7
Support Vector Machine (SVM)	A statistical learning algorithm, used for supervised ML and food quality solutions [34,59].	e1071	kernel = polynomial; γ=0.02, degree = 3

**Table 4:** Performance measures in the comparison of the methods and algorithms (RF, k-NN, J48 and SVM) over the cross-validation and prediction dataset.

Algorithm	Metric	Cross-Validation			Prediction		
		Traditional	SPP	SPPe	Traditional	SPP	SPPe
RF	Accuracy	90.00	91.00	100.00	90.00	95.00	95.00
	Precision	71.88	71.88	100.00	86.67	96.88	96.88
	Recall	68.93	69.43	100.00	86.67	90.00	90.00
	Time (s)	65.35 (±0.13)	281.63 (±1.09)	217.11 (±0.40)	62.53 (±0.12)	268.71 (±0.39)	207.07 (±0.34)
k-NN	Accuracy	77.56	70.56	95.56	80.00	60.00	75.00
	Precision	60.79	57.25	95.85	74.51	52.75	65.63
	Recall	58.79	53.88	94.81	66.67	53.33	63.33
	Time (s)	64.50 (±0.10)	279.34 (±0.94)	209.49 (±0.36)	62.44 (±0.15)	268.51 (±0.36)	206.11 (±0.29)
J48	Accuracy	89.00	88.00	100.00	85.00	85.00	100.00
	Precision	71.88	71.88	100.00	79.77	91.67	100.00
	Recall	68.43	67.93	100.00	83.33	70.00	100.00
	Time (s)	70.14 (±0.26)	353.37 (±2.31)	210.79 (±0.37)	62.61 (±0.10)	270.71 (±0.38)	206.32 (±0.33)
SVM	Accuracy	93.00	92.00	98.89	80.00	95.00	95.00
	Precision	70.42	72.50	99.11	89.47	96.88	96.88
	Recall	70.00	70.36	98.57	60.00	90.00	90.00
	Time (s)	64.62 (±0.15)	280.57 (±0.95)	213.40 (±0.43)	62.83 (±0.12)	268.66 (±0.37)	206.75 (±0.37)
